# Optimal Geostatistical Methods for Interpolation of the Ionosphere: A Case Study on the St Patrick’s Day Storm of 2015

**DOI:** 10.3390/s20102840

**Published:** 2020-05-16

**Authors:** Marek Ogryzek, Anna Krypiak-Gregorczyk, Paweł Wielgosz

**Affiliations:** Faculty of Geoengineering, University of Warmia and Mazury in Olsztyn, 10-719 Olsztyn, Poland; a.krypiak-gregorczyk@uwm.edu.pl (A.K.-G.); pawel.wielgosz@uwm.edu.pl (P.W.)

**Keywords:** ionosphere, TEC, GNSS, geostatistical methods, MPQE

## Abstract

Geostatistical Analyst is a set of advanced tools for analysing spatial data and generating surface models using statistical and deterministic methods available in ESRI ArcMap software. It enables interpolation models to be created on the basis of data measured at chosen points. The software also provides tools that enable analyses of the data variability, setting data limits and checking global trends, as well as creating forecast maps, estimating standard error and probability, making various surface visualisations, and analysing spatial autocorrelation and correlation between multiple data sets. The data can be interpolated using deterministic methods providing surface continuity, and also by stochastic techniques like kriging, based on a statistical model considering data autocorrelation and providing expected interpolation errors. These properties of Geostatistical Analyst make it a valuable tool for modelling and analysing the Earth’s ionosphere. Our research aims to test its applicability for studying the ionosphere, and ionospheric disturbances in particular. As raw source data, we use Global Navigation Satellite Systems (GNSS)-derived ionospheric total electron content. This paper compares ionosphere models (maps) developed using various interpolation methods available in Geostatistical Analyst. The comparison is based on several indicators that can provide the statistical characteristics of an interpolation error. In this contribution, we use our own method, the parametric assessment of the quality of estimation (MPQE). Here, we present analyses and a discussion of the modelling results for various states of the ionosphere: On the disturbed day of the St Patrick’s Day geomagnetic storm of 2015, one quiet day before the storm and one day after its occurrence, reflecting the ionosphere recovery phase. Finally, the optimal interpolation method is selected and presented.

## 1. Introduction

In recent decades, Global Navigation Satellite Systems (GNSS) have found several applications in a broad range of geosciences. Along with the ongoing development into GNSS technology, the offered accuracy has increased, opening up new research possibilities. GNSS signals are primarily applied to provide the user’s position [1,2]. This technology is also increasingly used to monitor deformations in the Earth’s crust [3]. However, precise GNSS positioning and GNSS-based geodetic and geodynamic studies require accurate corrections of ionospheric delay [4,5]. In this respect, another field of studies based on GNSS data is atmosphere remote sensing [6,7]. Therefore, satellite data are also often used in modelling the ionosphere. There are a number of various modelling methods that differ in terms of accuracy and reliability [8]. A comprehensive review of the most popular GNSS-derived ionosphere models is provided in [9,10].

A predominant group of GNSS-derived global ionospheric maps (GIMs) are elaborated by using mathematical methods [11], such as spherical harmonic expansion (SHE). This approach is often used for two reasons. Firstly, because of using noisy phase-smoothed pseudorange data. Secondly, it is often used due to the heterogeneous resolution of the observations. This approach reflects long-term effects of spatial and temporal changes of total electron content (TEC) well. However, it is characterized by a loss of information about the local structures in the ionosphere. The obtained ionosphere maps have low spatial and temporal resolution, and only allow for a general overview of the ionosphere response to a geomagnetic storm.

In order to overcome the irregular data problem, several authors have proven that the use of the kriging technique is also possible. This approach takes into account the spatial correlation among the data to be interpolated. Odijk [4] and Orus et al. [12] conducted research on the application of kriging technique to global ionosphere mapping. In Orus et al., ordinary kriging was applied to improve the ionospheric maps from the Technical University of Catalonia (UPC), computed with GPS data. The authors show that new UPC kriging GIMs (UQRG) are characterised by lower Root Mean Square (RMS) error in the observed slant TEC (sTEC) than the original UPC GIMs and IGS GIMs. However, this model remains at a resolution—namely, 2.5° × 5.0° × 15 min—that does not provide fine details of the ionosphere.

Kriging is increasingly used in regional ionosphere modelling. Stanislawska et al. [13] applied this technique in regional vertical TEC (vTEC) estimation over the European area. The analyses were carried out for a few quiet and disturbed days in September 1999. Other researchers also successfully used this interpolation technique in regional ionosphere studies. Deviren and Arikan [14] presented the mapping algorithm based on universal kriging with linear trend for midlatitude regions and ordinary kriging for other regions. According to Shukla et al. [15], for the Indian region, it is clearly more suitable to use ordinary kriging in place of planar fit to estimate the ionospheric delay. They also found out that ordinary kriging performed better than the bilinear interpolation technique.

Due to the dynamic nature of changes in the disturbed ionosphere, the accuracy and the resolution of the developed models is still often insufficient to analyze the storm time effects in detail [16] as well as to support precise positioning applications, especially those requiring fast ambiguity resolution [10]. Therefore, the development of high-accuracy models with higher spatial and temporal resolution is still a popular research topic.

In this contribution, we aim at providing examples of evaluation and validation of various TEC interpolation techniques—namely statistical and deterministic methods. Therefore, Geostatistical Analyst was used to provide a set of ionospheric grids that were subsequently evaluated in terms of their accuracy and reliability. Recently, a new approach based on processing precise carrier phase GNSS data was proposed by Krypiak-Gregorczyk et al. [17]. This approach results in precise ionospheric TEC estimations in an irregular cloud of ionosphere piercing points (IPP). This cloud of points has to be provided to users in the form of a regular grid. However, there is a large number of available interpolation methods, and it is difficult to choose the most suitable one that would be appropriate to a range of geographical regions and ionospheric conditions. Therefore, in this initial contribution, we aim to test different geostatistical methods offered by Geostatistical Analyst for modelling the ionosphere over the European region (e.g., ionospheric mid-latitudes). All the geostatistical methods available in the Geostatistic Wizard of ESRI ArcMap10.4, divided into deterministic and stochastic methods, were used in the work. Geostatistics is a group of algorithms, based on generalised least-squares regression, enabling solutions of both deterministic and stochastic models [18]. It has a solid mathematical theoretical foundation developed by Matheron [19,20]. An advantage of the deterministic method is the lack of any requirement as to the normality of the data distribution of the analysed feature, because it assumes a non-random character of the studied phenomenon and describes the model as one fixed function defined in space. In contrast, stochastic methods assume a random nature of the value of an environmental variable, which is a stationary Gaussian stochastic process [21,22]; as a result, krigings allow geostatistical simulations [23].

The applicability of geostatistical methods has been well documented in Earth Sciences [23,24,25]. However, it was only in the 1980s that these methods started to be applied on a more regular basis in disciplines other than geology, where they originate. Geostatistics is a branch of spatial data statistics that not only includes the attributes in the analysis, but also their location in space and/or time [26]. Its specific nature results from the fact that it analyses and models continuous variables (attributes), i.e., variables with a determined (though usually unknown) value at each point of the analysed field, or, in the case of qualitative variables, a determined state [27]. This method is founded on probability theory, specifically the random functions theory [28,29].

Since the early 1990s, geostatistics has mostly been used to describe spatial structure and to estimate the values of attributes in non-measured fields. Geostatistics takes into consideration the spatial distribution of attributes. It is used to analyse how attribute values depend on the distance between the points at which particular values have been measured. That is why it is easier to statistically describe and interpret the spatial distribution of an analysed attribute. We can distinguish geostatistical methods that make it possible to analyse not only quantitative, but also qualitative data. Detailed descriptions of the geostatistical methods applied in the research are available in the literature of the subject [23,24,30]. These methods are based on research conducted in mathematics and life sciences [20,22,31], hence they have solid theoretical bases. Moreover, geostatistical methods can be used to evaluate precisely the uncertainty of the estimates of the attribute value in areas where the attributes were not actually measured. Geostatistical Analyst is a set of advanced tools for analysing spatial data and generating surface models through the use of statistical and deterministic methods. It enables the interpolation of models on the basis of data measured at chosen points. Geostatistical Analyst enables analyses of data variability, setting data limits and checking global trends, as well as creating forecast maps, estimating standard error and probability, making various surface visualisations, including contour lines (isolines), and analysing spatial autocorrelation and correlation between multiple data sets. The data can be interpolated using deterministic methods providing surface continuity, and also by determining an estimation of the most probable value, while the kriging techniques, which are based on a statistical model considering data autocorrelation, evaluate the expected error. These properties of Geostatistical Analyst make it a valuable tool for modelling and analysing the Earth’s ionosphere. Hence, our research aims to test its applicability for studying the ionosphere, and ionospheric disturbances in particular. The need to analyse the applied interpolation methods and the effects of selecting the wrong interpolation methods for spatial data visualisations has been pointed out by Ogryzek [32]. The article uses the method of the parametric assessment of the quality of estimation (MPQE) proposed by Ogryzek [33]. Here, the validation is made on the basis of comparing methods by using root mean square prediction error (RMSE) in particular [30,34].

## 2. Methodology

For providing ionospheric TEC data for further analysis, dual-frequency multi-GNSS data (GPS + GLONASS) from the Polish active geodetic network (ASG-EUPOS) and European EUREF Permanent Network (EPN) stations were used [35]. The presented regional ionosphere model is computed using exclusively precise, absolute (non-differenced) carrier phase GNSS measurements, several orders of magnitude more precise than pseudorange ones. The geometry-free (LGF) carrier phase linear combination is used to eliminate geometry-related observational errors—receiver and GNSS satellite clock biases, tropospheric delays, etc. Unfortunately, this combination, along with information about ionospheric delays, also includes carrier phase ambiguities and carrier phase hardware delays that have to be estimated.

**Figure 1:**
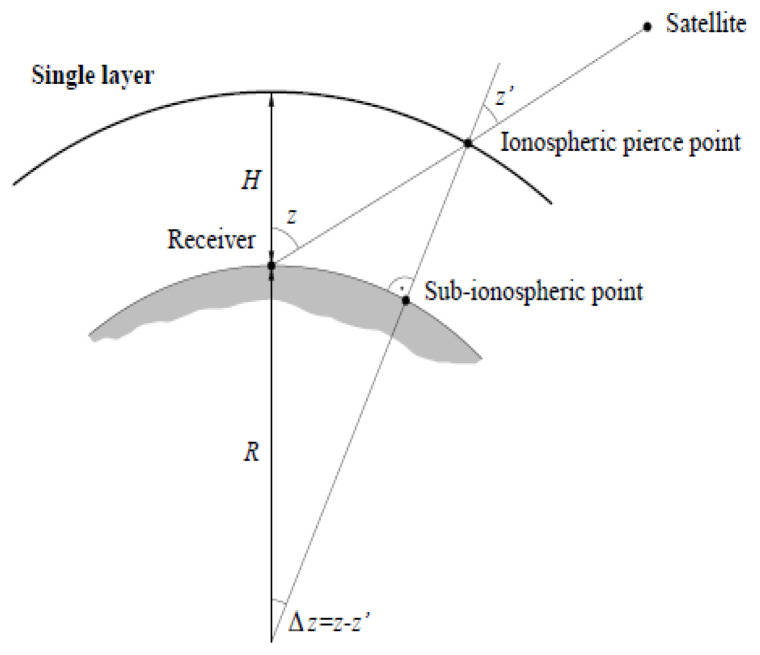
Single layer model geometry [36]. *z*-satellite’s zenith distance at the receiver’s location; *z*’-satellite’s zenith distance at the ionospheric pierce point; *R*—the mean Earth radius; *H*—the height of the single layer.

The University of Warmia and Mazury in Olsztyn (UWM) model used in this study is based on the single layer model (SLM) approach [36] (Figure 1). In the first processing step, carrier phase biases (ambiguities + hardware delays) are estimated.

For each continuous satellite arc [17]. In the second step, the obtained biases are used, together with dual-frequency, multi-GNSS carrier phase observations, to calculate the ionospheric TEC at IPP locations [37,38]. Then, at the third step, Geostatistical Analyst is used to provide the final ionospheric grid (map) for users.

**Figure 2:**
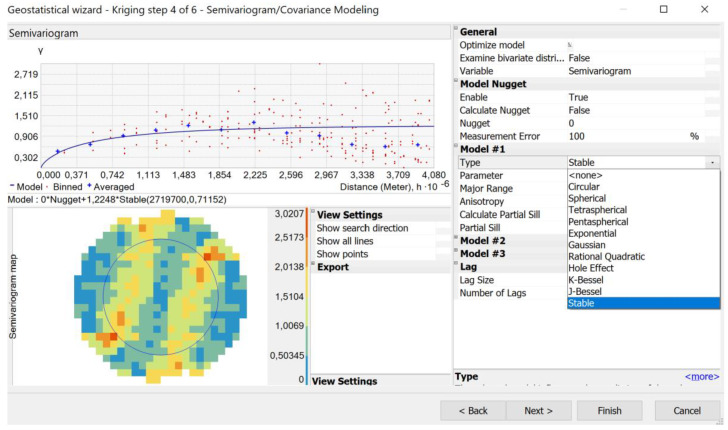
Geostatistical modelling in ArcMap software.

In the modelling process, the semivariogram of the approximated function has been studied using nugget, spherical, Gaussian, power, exponential and linear base models. We defined not only the range, but also the direction to specify the sector type (Figure 2). The choice of the appropriate base model was carried out using the MPQE approach proposed by Ogryzek [33], which is based on: ME (mean prediction error), RMSE (root mean square prediction error), ASE (average standard error), MSE (mean standardised prediction error), and RMSSE (root mean square standardised prediction error). In the present study, for the geostatistical methods the abovementioned errors received different weightings: RMSE = 60%, ME = 10%, ASE = 10%, MSE=10%, RMSSE = 10%.

Accurately matching the model to the source data may not generate the lowest errors, which is why it is important to validate the results prior to choosing the optimal model. In practice, choosing the best method is problematic, due to a large amount of quality statistics of the estimation and the lack of clear selection criteria [33]. The MPQE method uses an optimisation algorithm based on estimation parameters from the validated cross validation (CV) and subsets validation (SV) model. The indicators (parameters) that have been analysed are the characteristics of the statistical error of the interpolation: RMSE and ME for each tested interpolation model. For each sample (analysis day), the MPQE values are based on the data of 45% RMSE CV and 5% ME CV (parameters from the validated cross validation) and 45% of the RMSE SV and 5% ME SV data (parameters from the validated subsets validation (SV) model).

To choose the optimal method, we use a weighting scheme: 45% RMSE (CV) + 5% ME (CV) and 45% for RMSE (SV) 5% ME (SV), i.e., for each model:

- IDW = 0.45 × RMSE (100% data) + 0.05 × ME (100% data) + 0.45 × RMSE (90% data) + 0.05 × ME (90% data);
- GPI = 0.45 × RMSE (100% data) + 0.05 × ME (100% data) + 0.45 × RMSE (90% data) + 0.05 × ME (90% data);
- OK = 0.45 × RMSE (100% data) + 0.05 × ME (100% data) + 0.45 × RMSE (90% data) + 0.05 × ME (90% data).

where IDW is inverse distance weighting, GPI is global polynomial interpolation and OK is ordinary kriging. The method with the lowest weighted prediction error is the optimal method.

## 3. Experiment

In order to test the applicability of the Geostatistical Analyst tools to the ionosphere interpolation, a test period of three days, characterised by very different geomagnetic and ionospheric activity, was selected. This period includes three days, from 16 to 18 March 2015, where:

- 16 March is characterised by a regular state of the ionosphere with Ʃ*Kp* = 19;
- 17 March is a stormy day with dynamic TEC variations and a clear increase over Europe with Ʃ*Kp* = 48 [16];
- 18 March presents the recovery phase of the storm, with low TEC value and Ʃ*Kp* = 39. The observational dataset included:
- Dual-frequency carrier phase and pseudorange GPS + GLONASS data from:
  ○ 50 GNSS stations of the Polish ASG-EUPOS network,
  ○ 200 GNSS stations of the EPN network,
- Sampling interval: 60 s,
- Data elevation cut-off: 30 degrees.

**Figure 3:**
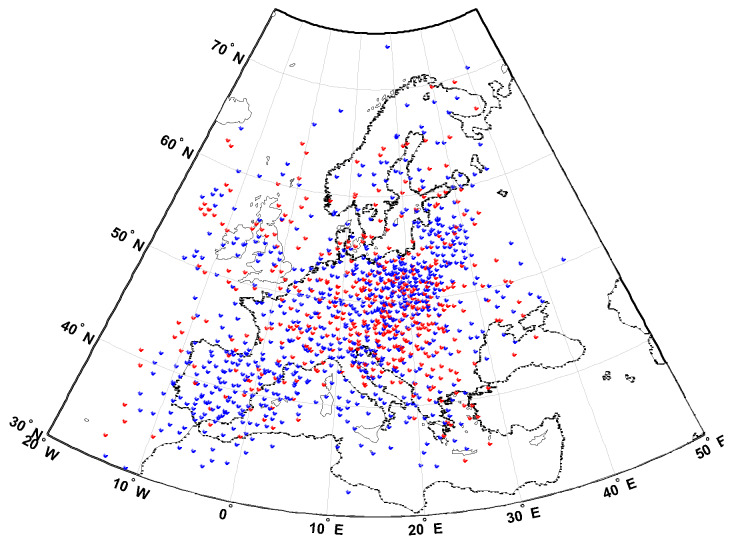
Examples of IPP locations at 11.10 UT on 17 March 2015 (red—IPPs for GPS; blue—IPPs for GLONASS).

Examples of IPP locations for their measurements collected at 11.10 UT on 17 March 2015 are presented in Figure 3.

## 4. Validation

Here, we aim to evaluate and validate various TEC interpolation techniques—statistical and deterministic methods. The procedure of validation of the map:

Stage 1: Preliminary data analysis;
Stage 2: Mapping by different interpolation methods;
Stage 3: Execution of validation;
Stage 4: Comparison of estimation assessment parameters;
Stage 5: Selection of the optimal geostatistical method.

The results obtained from the analyses (even comparing raw RMSE and ME from cross-validation) showed that the polynomial interpolation (LPI) method is the best among those tested, as the RMSE and ME values are a single study, although not always. It often happened that the OK method had better results than the LPI method. However, on average, LPI is the method that gives lower error values. Additionally, geostatic methods (krigings) require the implementation of a complex process, the analysis of errors of many models and the choice of the one that is closest to the criterion of estimation quality assessment. The choice of an appropriate model type in kriging methods is not unambiguous; the final decision is made by an analyst with extensive knowledge in this area. For example, for data during a storm (Table 1):

For each daily data set, we produced 72 maps at 20 min intervals. The validation was carried out to confirm, in a manner consistent with the assumptions, whether the applied interpolation procedures led to the expected results (Table 2).

It is clearly visible from Table 1 that, during the St Patrick’s Day storm, the MPQE increased by half. The lowest values were obtained one day after the storm. The worst results were obtained for global polynomial interpolation (GPI) and for simple kriging (SK). A day before the storm, the results are worse than the LPI results by 30–40%, on the day of the storm the difference is 70–80%, and one day after the storm it is more than 100–120%. The lowest MPQE was obtained on each day using the local polynomial interpolation (LPI) method. The MPQE value increased on the day of the storm, and then abruptly fell one day after the storm, during the recovery phase. The ordinary kriging (OK) method lies within +/- 0.05% statistical significance of MPQE, but only a day before and during the storm. On the day after the storm, the OK method is worse than the LPI method by 21%. This means that only the local polynomial interpolation method should be used after the storm, but in both calm and stormy periods either of the two methods (LPI or OK) can be used to model the ionosphere.

The average results for the analysis performed during the three tested days reveal that the best method was LPI, although each method has a statistically significant difference in results. Generally speaking, the results obtained for RMSE should not be averaged over several days, and modelling should not be made without recognising the RMSE errors. The differences in deviations from errors are at the level from 6% to 70%. The averaged MPQE results are close to errors obtained on a calm day before the storm, which makes it possible to conclude that LPI is the best method on calm days, even though the research undertaken on a calm day fails to show statistically significant difference of MPQE for the OK method.

**Figure 4:**
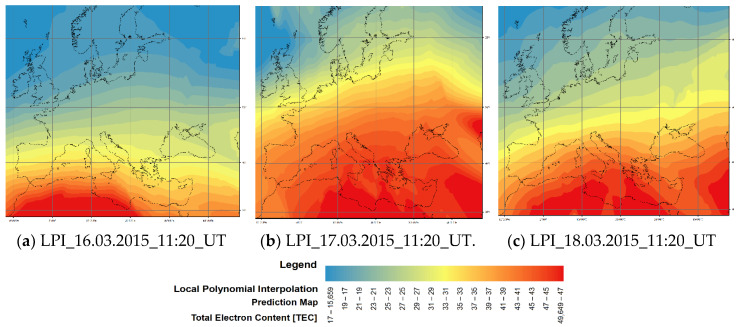
Ionosphere interpolation results during the St Patrick’s Day Storm (Source: Own study).

Figure 4 shows examples of the state of the ionosphere over Europe on 16 March 2015 (a), 17 March 2015 (b) and 18 March 2015 (c) at 11.20 UTC derived with the LPI method. A total of 72 maps were analysed for each interpolation method. According to Krivoruchko [18], RMSE is an indicator (parameter) recommended for comparing the results of spatial analysis using various interpolation methods. Its value should be as small as possible. For each sample (day of analysis), the MPQE (RMSE 100% data and RMSE 90% data) was the lowest if the LPI method was applied. In addition, errors generated during storms are the largest, while in calm periods, the errors drop on average by 65%. An important part of the analysis of the results is the analysis of partial results, where at times the OK method generated the smallest errors, or where the differences between errors generated in the OK and LPI methods were below the statistical significance. The similarities and differences in error generating in the ordinary kriging and polynomial interpolation methods, and the possibilities of obtaining similar errors as a result, have been described by Matheron [31]. Zhou et al. [25] suggested ordinary kriging as an alternative to estimation through the polynomial interpolation method.

**Figure 5:**
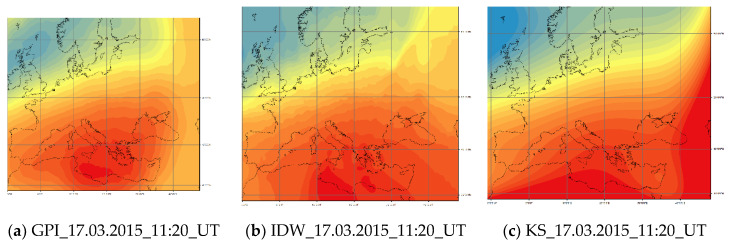

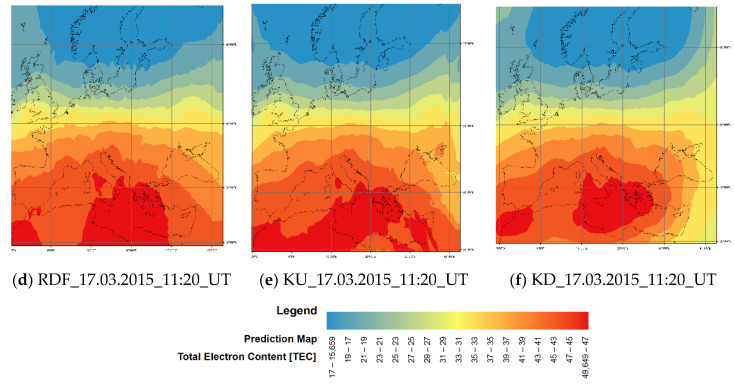
A comparison of ionosphere interpolation results during the St. Patrick’s Day Storm (Source: Own study).

Figure 5 shows a comparison for a stormy day at the same time for three different methods. The differences are clearly visible, which proves the need for model development and validation to select the most suitable one for a particular dataset.

## 5. Conclusions

Global maps of ionospheric total electron content are produced by interpolating GNSS-derived TEC measurements. These maps are produced to test data acquisition, monitoring facilities, and mapping techniques. The TEC mapping can provide accurate ionospheric calibrations to navigation systems. These maps are also used to monitor ionospheric weather, and to nowcast ionospheric storms, which often occur in response to activities in solar wind and the Earth’s magnetosphere and thermosphere. The objective of the presented analysis was to evaluate the accuracy of the interpolation methods, available in the analysis software ArcGIS 10.2.1, for the ionospheric TEC modelling for the area of Europe. Then, a number of interpolation methods were tested, both deterministic—inverse distance weighting (IDW), global polynomial interpolation (GPI), radial basis function (RBF), local polynomial interpolation (LPI), and geostatistical—ordinary kriging (OK), simple kriging (SK), universal kriging (UK) and disjunctive kriging (DK). The result is 72 maps per day representing the state of the ionosphere for the area of Europe. The interpolation precision indicates the average size of the resulting errors. Based on the case studies conducted, among the deterministic methods, the most accurate method is characterised by LPI, while among the geostatistical methods it is OK. This may suggest that the ionospheric TEC provided in an irregular cloud of IPPs is a process that is determined by the ionospheric physical properties, and to a lesser extent by random factors. However, when analysing quiet days only, the results show that geostatistical models may perform better. It means that one may choose different interpolation methods when modelling the quiet and disturbed ionosphere. The results obtained from the analyses (even comparing raw RMSE and ME from cross- and subset validation) showed that the LPI method, among the subjects, generates the lowest errors. It often happened that the OK method had better results than the LPI method. However, on average, LPI is the method that gives lower error values. Additionally, geostatic methods (kriging) require the implementation of a complex process, the analysis of errors of many models, and the selection of the one that is closest to the criterion of estimation quality assessment. The choice of an appropriate model type in kriging methods is not unambiguous; the final decision is made by an analyst with extensive knowledge in this area. For example, during the modelling process they have to perform many iterations for one series of data to compare results obtained by different approximation methods and (several) functions. However, in some cases, especially after the end of the storm, lower RMSE and ME parameters could be obtained by cracking methods. Nevertheless, taking into account that geostatistical methods require additional knowledge from the data analyst and should rather be used on smaller plots and require more time and, in addition, that the results do not show significant statistical differences, the LPI can be considered as the optimal method, where selecting only a number of sectors is a substitute for geostatistical modelling. Since this is an initial study, more focus should be made on the validation methods in the future. In the further analysis, interpolation methods should be looked at using a control set (validation layer) due to the large number of observations (every 20 min), or by using the cross-validation method of the leave-one-out type, consisting in the sequential elimination of data from each point and, performing interpolation at this point on the basis of the remaining data.

## Figures and Tables

**Table 1:** Test results of accuracy analysis of different interpolation methods [TECU].

	**100% of Data**		**90% of Data**		
**Day 75_6 am**	**ME**	**RMSE**	**ME**	**RMSE**	**MPQE**
Inverse distance weighting	0.013	0.545	0.0121	0.551	0.494
Global polynomial interpolation	0.001	0.999	0.001	1.013	0.905
Radial basic functions	0.005	0.587	0.003	0.627	0.546
Local polynomial interpolation	−0.015	0.472	−0.016	0.478	**0.429**
Kriging ordinary	−0.001	0.476	−0.002	0.479	0.430
Kriging simple	−0.029	0.530	−0.030	0.535	0.482
Kriging universal	−0.001	0.476	−0.002	0.479	0.430
Empirical Bayesian kriging	0.001	0.487	0.001	0.489	0.439
	**100% of Data**		**90% of Data**		
**Day 76_6 am**	**ME**	**RMSE**	**ME**	**RMSE**	**MPQE**
Inverse distance weighting	0.006	0.540	0.005	0.547	0.490
Global polynomial interpolation	0.001	1.094	0.000	1.103	0.989
Radial basic functions	0.002	0.517	0.001	0.524	0.469
Local polynomial interpolation	0.001	0.470	−0.001	0.478	**0.426**
Kriging ordinary	−0.001	0.474	−0.001	0.481	0.430
Kriging simple	0.169	0.573	0.174	0.590	0.540
Kriging universal	−0.001	0.474	−0.001	0.481	0.430
Kriging disjunctive	−0.002	0.489	−0.001	0.496	0.443
	**100% of Data**		**90% of Data**		
**Day 77_6 am**	**ME**	**RMSE**	**ME**	**RMSE**	**MPQE**
Inverse distance weighting	−0.023	0.359	−0.025	0.369	0.330
Global polynomial interpolation	−0.001	1.635	−0.000	1.614	1.4624
Radial basic functions	−0.006	0.355	−0.005	0.359	0.322
Local polynomial interpolation	0.039	0.255	0.029	0.257	0.234
Kriging ordinary	0.006	0.249	0.006	0.255	**0.227**
Kriging simple	0.388	0.673	0.392	0.691	0.653
Kriging universal	0.006	0.249	0.006	0.255	**0.227**
Kriging disjunctive	0.004	0.258	0.003	0.263	0.235

**Table 2:** A comparison of geostatistical methods, green– best results, red– worst results.

Date	Data Samples	Method	MPQE [TECU]
16.03.2015	19,650	LPI	0.51
		OK	0.52
		RBF	0.57
		UK	0.58
		IDW	0.60
		DK	0.61
		SK	0.65
		GPI	0.71
17.03.2015	18,900	LPI	0.80
		OK	0.81
		IDW	0.91
		UK	0.94
		DK	1.02
		RBF	1.08
		SK	1.39
		GPI	1.44
18.03.2015	21,350	LPI	0.28
		OK	0.34
		IDW	0.38
		RBF	0.44
		UK	0.44
		KD	0.45
		GPI	0.57
		SK	0.60

(Source: Own study). List of abbreviations: Inverse distance weighting (IDW); global polynomial interpolation (GPI); radial basis function (RBF); local polynomial interpolation (LPI); and geostatistical – ordinary kriging (OK); simple kriging (SK); universal kriging (UK); and disjunctive kriging (DK).
